# Engineering Highly Emissive Tetra(t‐Butyl)rubrene/off‐Stoichiometry Thiol‐Ene Hybrids Toward Flexible Luminescent Solar Concentrator‐Integrated Photovoltaics with Excellent Stability

**DOI:** 10.1002/smsc.202400121

**Published:** 2024-04-05

**Authors:** Yi Zhang, Zida Zheng, Zhixing Gan, Rui Huang, Xiaowei Zhang

**Affiliations:** ^1^ Department of Electrical Engineering and Computer Science Ningbo University Ningbo Zhejiang 315211 China; ^2^ School of Materials Science and Engineering Hanshan Normal University Chaozhou Guangdong 521041 China; ^3^ Center for Future Optoelectronic Functional Materials Nanjing Normal University Nanjing Jiangsu 210093 China; ^4^ National Laboratory of Solid State Microstructures Nanjing University Nanjing Jiangsu 210093 China

**Keywords:** flexibility, luminescent solar concentrator integrated photovoltaics, Monte Carlo ray‐tracing, tetra(t‐butyl)rubrene

## Abstract

Luminescent solar concentrators (LSCs) are highly valued in transparent photovoltaics for their versatility and adaptability as effective large‐area sunlight collectors. However, constructing LSCs as efficient, long‐term stable, and easily malleable power‐generating units still remains a challenge. Herein, high photostability, high photoluminescence quantum yield (*Φ*_PL_), and Stokes‐shifted emission are achieved in tetra(t‐butyl)rubrene/off‐stoichiometry thiol‐ene (TBRb/OSTE) hybrids by incorporating TBRb molecules into OSTE polymers. To demonstrate their potentials in LSCs, a luminescent solar concentrator integrated photovoltaics (LSCIPV) is created using a one‐step synthesis that combines the preparation of TBRb/OSTE hybrids, coupling with the silicon solar cell, and the encapsulation of both components. The LSCIPV demonstrates remarkable flexibility and the integration of an ethylene tetrafluoroethylene film contributes to a hydrophobic surface and antireflection effect, resulting in an external photon efficiency (*η*_ext_) of 4.9% and a power conversion efficiency of 1.04% for the 100 cm^2^ device. The LSCIPV exhibits good photostability over 800 h of UV light exposure. Monte Carlo ray‐tracing simulations indicate that an optimized TBRb‐based LSCIPV can potentially achieve an *η*_ext_ of 1.3%, even for a large device with an area of 1 m^2^.

## Graphical abstract

Tetra(t‐butyl)rubrene/off‐stoichiometry thiol‐ene hybrids exhibit excellent photostability, photoluminescence quantum yield, and Stokes‐shifted emission. Integrated in a one‐step synthesis, they form a flexible luminescent solar concentrator integrated photovoltaics system with an external photon efficiency of 4.9%. This system benefits from an antireflection effect and a hydrophobic surface for self‐cleaning. Furthermore, it demonstrates good photostability over 800 h.

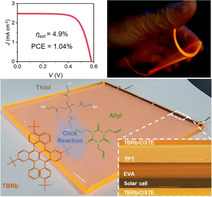

## 1 Introduction

Global climate change poses a threat to human survival, prompting the need for clean energy alternatives to traditional sources. However, limited urban space and tall buildings hinder the implementation of photovoltaics (PV) due to suboptimal sunlight exposure.^[^ ^1^, ^2^ ^]^ In the face of this challenge, luminescent solar concentrators (LSCs) have emerged as an ideal solution for building‐integrated photovoltaic (BIPV) applications.^[^ ^3^, ^4^, ^5^, ^6^ ^]^ LSCs can harvest both direct and diffuse sunlight, enabling effective conversion of solar energy into electricity and making them highly suitable for urban environments.^[^ ^7^ ^]^ LSCs employ luminophores embedded in a transparent polymer matrix to absorb incident light, enabling the directed concentration of the luminescence toward the edges for conversion into electricity by PV cells. Recently, flexible LSCs have attracted attention due to their potential applications in versatile areas, such as boat sails, tents, phone cases, and self‐powered flexible electronic devices.^[^ ^8^, ^9^, ^10^ ^]^ Meinardi et al. introduced flexible LSCs based on Si nanocrystals (NCs) embedded in a poly(lauryl methacrylate) (PLMA) matrix.^[^ ^11^ ^]^ The LSC achieved a high optical efficiency of 2.85% for a 12 × 12 cm^2^ device area, as the reabsorption loss was effectively minimized with the optimal Si NCs’ optical profile. Importantly, the flexible Si NCs‐based LSCs demonstrated consistent optical output across all angles (from 0° to 180°), making them suitable for applications in architectural designs requiring complex curvatures. Cai et al. have proposed a highly soft LSC by embedding highly emissive Mn^2+^/Yb^3+^ codoped CsPbCl_3_ NCs in a polydimethylsiloxane (PDMS) matrix.^[^ ^12^ ^]^ Benefiting from the quantum cutting effect of the NCs and the reduced C–H overtone absorption of the PDMS polymers, the LSC achieved an external photon efficiency (*η*_ext_) of 7.3% for the device size of 13 × 13 × 0.5 cm^3^.

Although there has been notable progress in enhancing the performance of flexible LSCs,^[^ ^11^, ^12^, ^13^, ^14^ ^]^ there remain several challenges that hinder their practical application. NCs have emerged as the most common option for LSCs due to their ability to provide tunable and Stokes‐shifted emission. However, existing NCs designed for LSCs have limitations, such as moderately low photoluminescence quantum yield (*Φ*_PL_) and small absorption cross‐sections.^[^ ^15^, ^16^, ^17^ ^]^ Current flexible LSCs predominantly employ PDMS or PLMA transparent flexible polymers as the matrices. The long side chains of PLMA can prevent agglomeration of NCs, meanwhile, low optical absorption in the visible spectrum can minimize the luminescence loss in the waveguide.^[^ ^11^, ^15^, ^18^ ^]^ However, PLMA has been identified as one of the most susceptible poly(alkyl methacrylate) to both thermal and photo degradation, which undermines the long‐term performance of LSCs.^[^ ^19^, ^20^, ^21^ ^]^ PDMS possesses favorable properties such as mechanical resistance, chemical resistance, and thermal stability. Optically, PDMS exhibits high transparency and a low refractive index of 1.41, resulting in a minimal reflection of ≈ 2.9%.^[^ ^22^, ^23^, ^24^, ^25^, ^26^ ^]^ These characteristics position PDMS as a strong candidate for LSC matrices.^[^ ^10^, ^12^, ^27^ ^]^ However, PDMS is known to allow the permeation of gas and moisture to degrade the embedded lumiphores, rendering it unsuitable for long‐term usage.^[^ ^28^ ^]^

Tetra(t‐butyl)rubrene (TBRb) as a yellow fluorescent emitter has been widely employed to design high‐efficiency organic light‐emitting diodes (OLEDs) for its excellent luminescence properties, high carrier mobility, high absorption coefficients, high radiative rate, and resistance to concentration quenching.^[^ ^29^, ^30^ ^]^ An external quantum efficiency (EQE) as high as 25% has recently been achieved in TBRb‐doped OLEDs.^[^ ^31^ ^]^ These excellent properties endow TBRb with the quality to replace NCs in the LSC. However, several obstacles hinder the construction of high optical efficiency LSCs based on TBRb. First, the luminescent properties of TBRb are detrimentally influenced by the reaction with molecular oxygen when exposed to light.^[^ ^32^, ^33^ ^]^ Second, when TBRb is dissolved in a solution, its *Φ*_PL_ is relatively lower.^[^ ^34^ ^]^ Here, we present TBRb/off‐stoichiometry thiol‐ene (OSTE) hybrids, in which TBRb is incorporated into the OSTE polymers using the thiol‐ene click reaction. Oxygen cross‐sensitivity of TBRb is eliminated by employing OSTE polymers as an oxygen‐scavenger. Additionally, by altering the dipole moment of TBRb, the OSTE polymers alter the PL characteristics of TBRb, leading to a reduction in the reabsorption effect. Furthermore, both steady‐state and time‐resolved PL spectroscopic studies reveal the OSTE polymers enhance the *Φ*_PL_ of TBRb through impeding nonradiative recombination. Leveraging the significant improvement in the optical performances of TBRb conferred by the OSTE polymers, we develop a facile synthesis method for the luminescent solar concentrator integrated photovoltaics (LSCIPV). This method combines the three elements into a single step, encompassing the TBRb‐based LSC preparation, the coupling between the LSC and the silicon solar cell, and the encapsulation of both the solar cell and the LSC. This unique flexible LSC system provides an antireflection (AR) effect, resulting in a ≈5% improvement in power conversion efficiency (PCE), while also featuring a hydrophobic front surface for self‐cleaning dust and pollutants. The LSCIPV also shows good photostability with minimal degradation over 800 h of continuous 10 W UV light irradiation.

## 2 Results and Discussion

**Figure 1:**
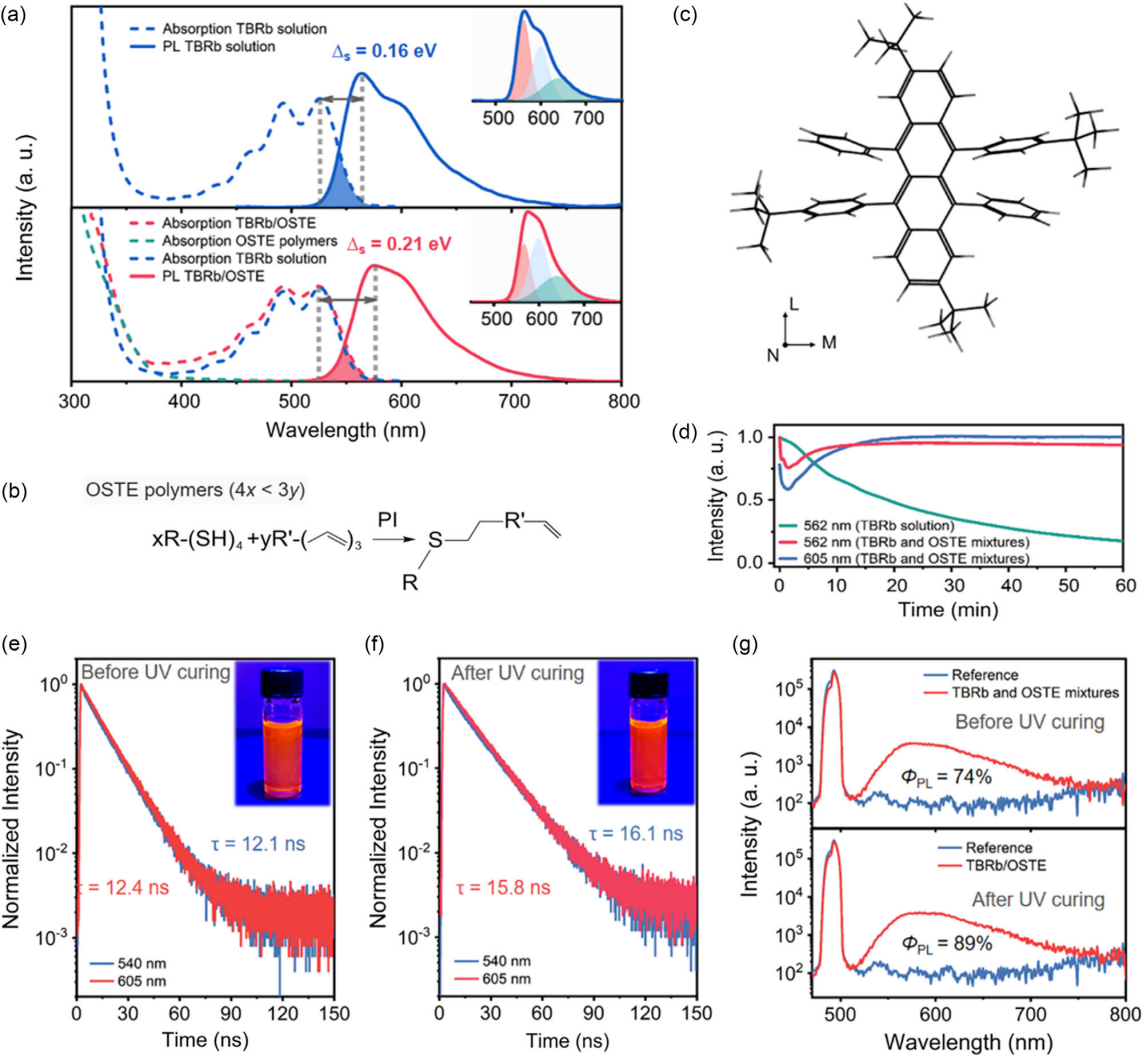
a) Absorption and PL spectra of TBRb dissolved in toluene (blue lines) and TBRb/OSTE hybrids (red lines). The absorption spectrum of OSTE polymers is plotted by green dashed lines. The insets show the peak fitting on the corresponding PL spectra. b) Reaction mechanism of radical thiol‐ene coupling in OSTE polymers. *xR–(SH)*_4_ and *yR’–(CH*_2_ *–CH = CH*_2_ *)*_3_ are thiol and allyl monomers. In OSTE polymers, off‐stoichiometric formulations of thiol‐enes with an excess of allyl functional groups (where 4x < 3y) result in a polymer with residual unreacted allyl anchor groups. c) The molecular structure of TBRb. d) The luminescence intensity as a function of UV light exposure duration for TBRb in solution and for TBRb and OSTE mixtures, respectively. PL decay curves of the e) TBRb and OSTE mixtures and f) the TBRb/OSTE hybrids obtained at an excitation wavelength of 460 nm. The insets show the photographs of e) the TBRb and OSTE mixtures and f) the TBRb/OSTE hybrids under 460 nm light. g) PL spectra for photoluminescence quantum yield (*Φ*_PL_) measurements in the absence and presence of the TBRb and OSTE mixtures (top) or the TBRb/OSTE hybrids (bottom) under 460 nm excitation.

As shown in **Figure** **1**a, the TBRb solution exhibits a main absorption band beyond 350 nm and two weak absorption bands at 490 and 525 nm. The main absorption is caused by the excitation from the highest occupied molecular orbital to the lowest unoccupied molecular orbital.^[^ ^35^ ^]^ After immobilization by the OSTE polymers (Figure 1b and Note S1, Supporting Information), the TBRb/OSTE hybrids show the clear combinatorial absorption profile of the two constituents. Both PL spectra of the solution and hybrid contain three PL subbands at 565, 605, and 640 nm (Figure 1a). The band at 565 nm is driven by the dipole moment M along the c‐axis, and the band at 605 nm is driven by the dipole moment L along the b‐axis (Figure 1b).^[^ ^36^ ^]^ The energy gap between the two bands at 565 nm (2.19 eV) and 605 nm (2.05 eV) is about 0.14 eV, which is attributed to C–C stretching vibration. Upon immobilizing TBRb with OSTE polymers, the decrease in the intensity ratio between the 565 nm and 605 nm bands suggests that the dipole moments of TBRb are potentially influenced by the thiol‐ene click reaction. Consequently, the Stokes shift (Δ_s_) is enlarged from 0.16 to 0.21 eV. The Δ_s_ for TBRb originates from unresolved vibrational progressions involving low‐frequency modes that are characterized by appreciable displacements in the excited state.^[^ ^37^ ^]^ To gain insights into the variation of the dipole moment of TBRb before and after the thiol‐ene click reaction, density functional theory (DFT) is utilized for computation. We employed the B3LYP hybrid functional method within DFT, and selected the 6‐31 G(d) basis set in the Gaussian 09 packet. The calculated dipole moment of TBRb is 0.135 Debye in thiol monomers, 0.146 Debye in allyl monomers, and 0.188 Debye in the OSTE polymers. It is important to note that the Stokes shift is not always well correlated with performance, as the reabsorption losses are determined by the specific overlap profiles between the absorption and PL spectra and the path length to the LSC edge. Therefore, we employ the modified overlap integral (OI*), defined as the ratio of the overlap integral of normalized absorption and PL spectra to the integral of the normalized PL spectrum, to estimate the reabsorption losses in the TBRb/OSTE hybrids. The OI* of the TBRb/OSTE hybrids is calculated to be 0.028, indicating a low probability of reabsorption compared to other organic luminophores that serve as emitters in the LSC (see details in Note S2, Supporting Information).

To further explore how the thiol‐ene click reaction plays a role in the PL of TBRb, we investigated the temporal luminescence dynamics of the TBRb and OSTE mixtures (Figure 1c), which contain TBRb, thiol monomers, allyl monomers, photo‐initiators (PIs), and N, N‐dimethylformamide (DMF). The PL at 565 and 605 nm drops sharply and reaches the minimum at 1.5 mins due to the exothermic behavior of thiol‐ene click reaction, which enhances the nonradiative transition. The PL at 565 and 605 nm subsequently recovers and reaches a constant value at the end of the thiol‐ene click reaction since the temperature of the TBRb/OSTE hybrids gradually returns to room temperature. After the thiol‐ene click reaction, the PL at 565 nm decreases while the emission at 605 nm increases, resulting in a smaller overlap between the absorption and PL spectra (Figure 1a). Consequently, the PL of the TBRb/OSTE hybrids (red solid line in Figure 1c) remains stable under UV light irradiation, whereas the PL of the TBRb solution (green solid line in Figure 1c) experiences a gradual decay. Given that UV or visible light can cause molecular oxygen to react with TBRb, OSTE polymers are considered as an oxygen scavenger to prevent the reaction, thereby ensuring the stability of TBRb in the sunlight.^[^ ^38^, ^39^ ^]^

**Figure 2:**
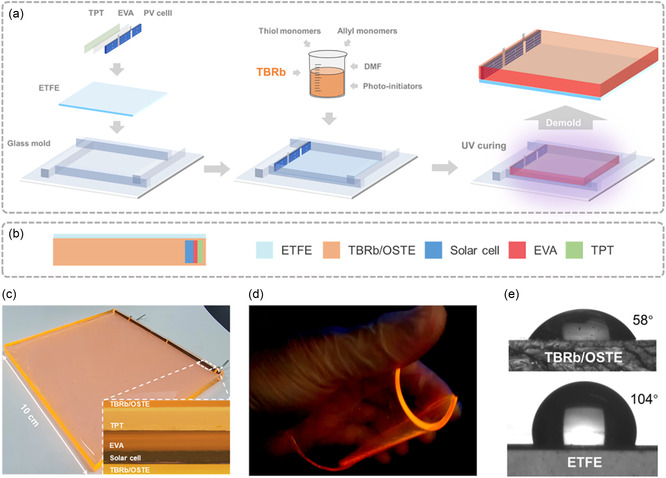
a) Schematic representation of the fabrication process of the LSCIPV based on TBRb/OSTE hybrids. b) Diagram of the LSCIPV. c) Photograph of the LSCIPV under ambient light. Inset is a confocal microscope image of the internal composition of the LSCIPV. d) Photograph of a flexible LSCIPV under UV illumination. e) The water contact angles of TBRb/OSTE hybrids (top) and the ETFE film (bottom).

The time‐resolved PL decay curves of the TBRb and OSTE mixtures (Figure 1d) and the TBRb/OSTE hybrids (Figure 1e) were measured and fitted by biexponential functions. The average lifetimes of the TBRb and OSTE mixtures solution monitoring at 540 and 605 nm are 12.1 and 12.4 ns, respectively. After curing, the average lifetimes of the TBRb/OSTE hybrids monitoring at 540 and 605 nm are 16.1 and 15.8 ns, respectively. The lifetimes of the bands at 565 and 605 nm are nearly the same, in consistency with the fact that these two bands are split by C–C stretching vibration. **Figure** **2**f shows that the *Φ*_PL_ of the TBRb and OSTE mixtures, after UV curing, increases from 74% to 89%, surpassing that of most NCs (see details in Table S2, Supporting Information). Additionally, the temperature‐dependent time‐resolved PL decay curves reveal a substantial decrease in the lifetime of the TBRb and OSTE mixtures with increasing temperature, whereas the lifetime of the TBRb/OSTE hybrids remains constant (see details in Figure S3, Supporting Information). This suggests that the observed increase in both the lifetimes and *Φ*_PL_ for TBRb immobilized by the OSTE polymers is probably due to the steric hindrance effect.^[^ ^40^ ^]^ The four substituted phenyl rings of the TBRb attached to the two internal rings dynamically rotate and vibrate against the stator on the single bond axes (Figure 1b). The vigorous intramolecular motions are nonradiative energy‐consuming. The flexibility of the TBRb enables the phenyl rings to dynamically rotate, bend, or vibrate in the solution and uncured mixtures, which serve as nonradiative relaxation channels for the excited state to return to the ground state, whereas for TBRb immobilized by the OSTE polymers, owing to the physical constraint associated with the space limitation, the intramolecular motions are restricted. Consequently, the rapid nonradiative channel is blocked, leading to the increased PL lifetime and *Φ*_PL_.

It is clear from the above results that OSTE polymers can simultaneously improve the photostability and PL efficiency of TBRb. Therefore, OSTE polymers are used as encapsulation materials for TBRb to design a LSCIPV. Figure 2a illustrates a schematic representation of the TBRb‐based LSCIPV and a description of the preparation procedure. Briefly, a laser cutting process is used to size the commercial silicon PV cell to fit the edge of the LSC. The back of the silicon PV cell is attached to the corrosion‐resistant Tedlar Polyester Tedlar (TPT) backsheet using the ethylene vinyl acetate (EVA) under vacuum. An ethylene tetrafluoroethylene (ETFE) film is placed on the inner bottom surface of the glass mold. Then the cell is erected on the film at a position close to the inner surface of the glass mold. The TBRb and OSTE mixtures are subsequently poured into the glass mold. The entire preparation platform is irradiated by UV light for 10 min under vacuum. Finally, the glass mold is detached carefully, and the LSCIPV is obtained (Figure 2b). Here, OSTE polymers not only act as an encapsulation material for TBRb, but also directly couple the PV cell without any adhesive, as shown in Figure 2c. The photograph of the fabricated LSCIPV under UV irradiation shows a high softness being easily bent to a semi‐cylindrical configuration (Figure 2d). Moreover, the performance of the LSCIPV exhibits a minimal decrease even after 300 bending cycles (see details in Note S5, Supporting Information). A strong orange emission from its edges demonstrates its potential for realizing curved or flexible BIPV elements.

The ETFE film is widely used as the front sheet to encapsulate flexible solar cell modules due to its excellent UV resistance, high transmittance, and outstanding tensile strength.^[^ ^41^, ^42^ ^]^ Moreover, the ETFE film shows the hydrophobic property with a water contact angle (CA) of 104° due to the large bond energies of C—F and long perfluoroalkyl side chains (Figure 2e), while TBRb/OSTE hybrids exhibit the hydrophilic native surface properties of OSTE polymers (CA = 58° in Figure 2e). The integration of an ETFE film into the LSCIPV structure enhances the hydrophobic properties of the front surface of the device. This improvement diminishes water retention on the device's surface, thereby reducing both light reflection and light scattering. Consequently, this allows a greater amount of light to enter the LSCIPV.

**Figure 3:**
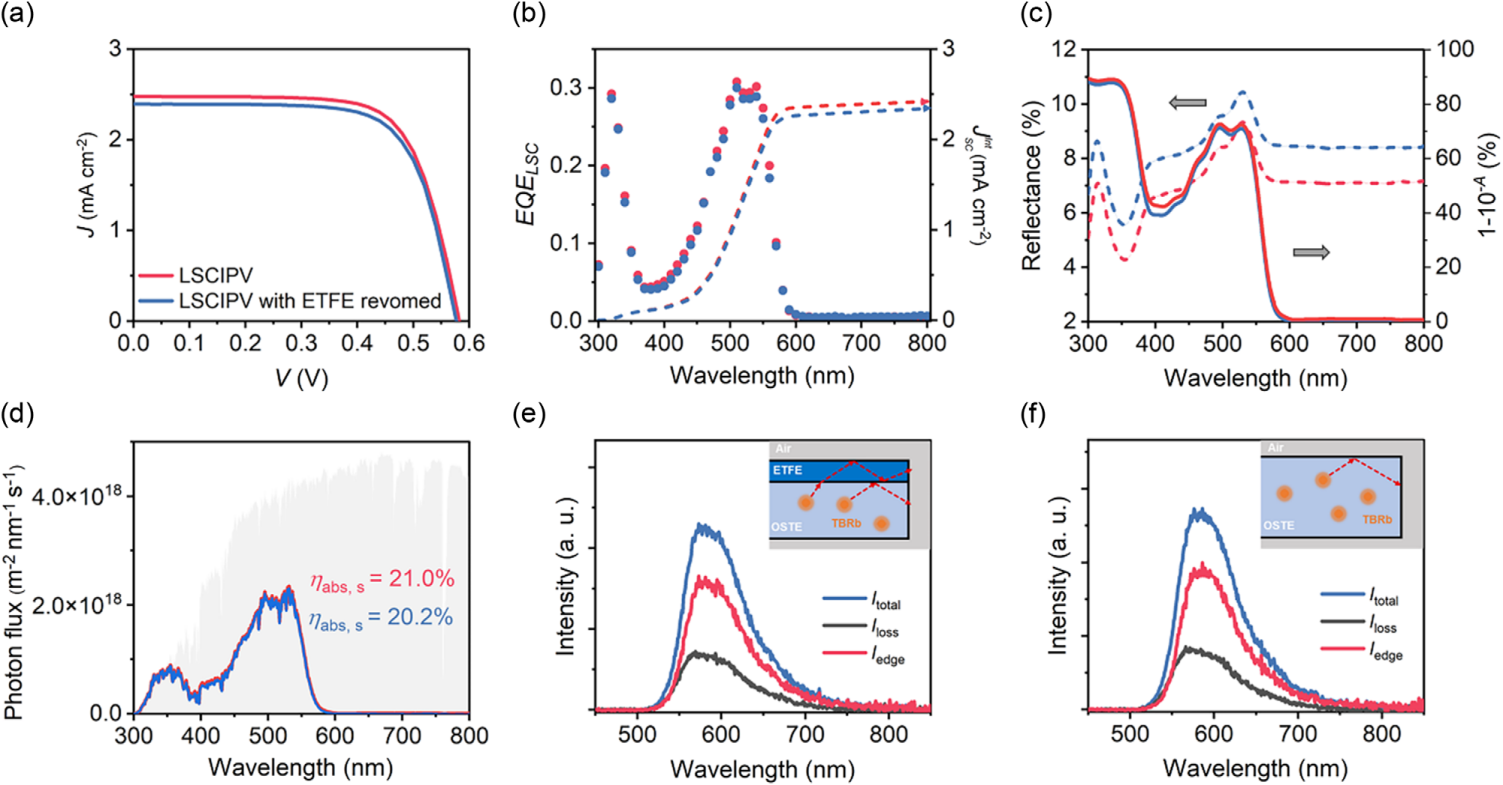
a) Current density versus voltage (*J*–*V*) characteristics, b) external quantum efficiency (*EQE*_LSC_) spectra, c) reflectance and quasi‐absorption (1‐10^−*A*^) spectra, and d) calculated solar absorption (*η*_abs,s_) for the LSCIPV (red) and the same one without the ETFE film (blue). The AM 1.5 G photon flux spectrum is shown in (d) by gray shading. e) PL spectra for the unmasked LSCIPV (blue) and the edge‐masked LSCIPV (gray). The edge PL spectrum (red) is obtained by subtracting the above two spectra. Inset is the diagram of the total internal reflection of radiative photons within the LSCIPV. f) The same PL measurements and diagram as (e) for the LSCIPV without the ETFE film.

**Figure 4:**
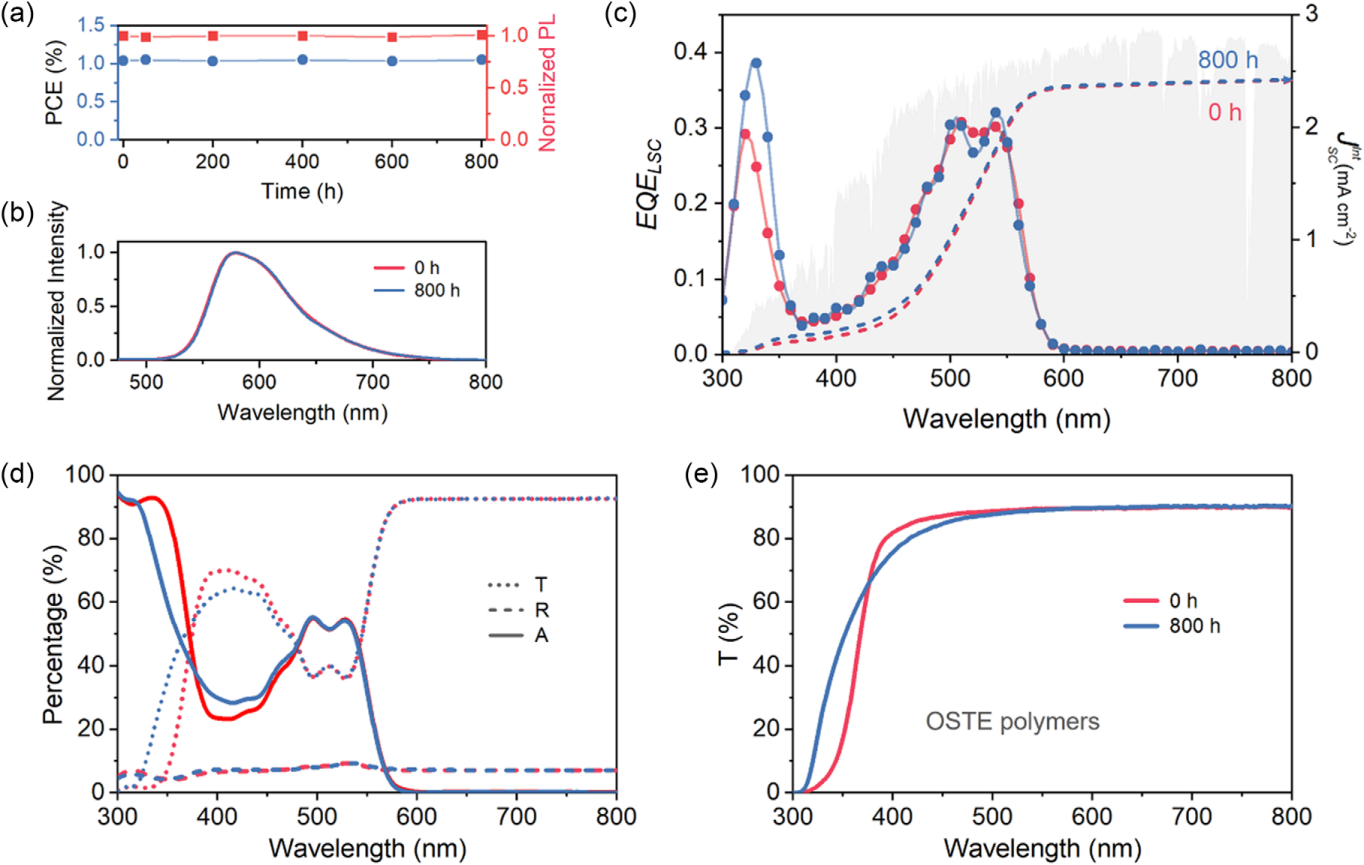
a) PCE and normalized integrated PL intensity for the LSCIPV measured under AM 1.5 G solar illumination as a function of UV light exposure time. b) PL spectra, c) *EQE*_LSC_ spectra, and d) transmittance (*T*), reflectance (*R*), and absorption (*A*) spectra for the LSCIPV before and after 800 h UV light exposure. The AM 1.5 G photon flux spectrum is shown in (d) by gray shading. e) *T* spectra for OSTE polymers before and after 800 h UV light exposure.

In addition to being used as a protective front sheet, the ETFE film also improves the solar concentration performances via the AR effect. As shown in **Figure** **3**a, the current density versus voltage (*J*–*V*) characteristic of the LSCIPV shows a short‐circuit current density (*J*_sc_) of 2.48 ± 0.06 mA cm^−2^, open‐circuit voltage (*V*_oc_) of 0.58 ± 0.01 V, and fill factor (FF) of 72 ± 1%, resulting in a PCE of 1.04 ± 0.06%. As the ETFE film detached from the LSCIPV, *J*_sc_ decreases to 2.39 ± 0.05 mA cm^−2^ with similar *V*_oc_ and FF, and the PCE decreases to 0.99 ± 0.05%. Figure 3b shows that integrated *J*_sc_ ($J_{\text{sc}}^{\text{Int}}$) values calculated from EQE (*EQE*_LSC_(*λ*)) values match well with the *J*_sc_ from the *J*–*V* curves.^[^ ^43^, ^44^ ^]^ The *EQE*_LSC_ bands show a close resemblance to the absorption spectrum of TBRb/OSTE hybrids. The presence of a weaker *EQE*_LSC_ band in the UV region is attributed to the nonradiative relaxation of the OSTE polymers. The intensity of *EQE*_LSC_(*λ*) decreases when the ETFE film is detached from the LSCIPV. The reflectance (*R*(*λ*)) spectra shown in **Figure** **4**c reveal that the integration of an ETFE film within the LSCIPV structure imparts an AR effect, which reduces the reflectivity by 1.5%. Consequently, the integration of the ETFE film enhances the absorption of the LSCIPV and the average visible transparency from 74.5% to 75.3%, as shown in Figure 3c and Figure S5, Supporting Information. To precisely quantify the extent of improved absorption facilitated by the ETFE film, the absorption (*A*(*λ*)) spectrum (solid lines in Figure 3c) is convoluted with the AM 1.5 G photon flux solar spectrum (gray shading in Figure 3d). The solar absorption (*η*_abs,s_) is then determined as the ratio of the integrated areas of the solar absorption and the solar spectrum (Figure 3d). The *η*_abs,s_ is improved from 20.2% to 21.0% upon the integration of the ETFE film within the TBRb‐based LSCIPV structure. The AR effect observed in LSCIPV upon the integration of an ETFE film is attributed to a gradient distribution of refractive index along the direction of incident sunlight (see details in Note S7, Supporting Information). Given that the ETFE film can reduce the reflection of solar photons on the LSCIPV, it becomes essential to investigate whether it also impacts the edge output of the radiative photons. The edge emission efficiency (*η*_edge_) measured using an integrating‐sphere‐equipped spectrometer is defined as follows:^[^ ^45^, ^46^ ^]^

(1) $$\eta_{\text{edge}}=I_{\text{edge}}/I_{\text{total}}$$

where *I*_edge_ is the integral intensity of the edge PL band, and *I*_total_ is the integral intensity of the total PL band, as shown in Figure 3e,f. When the ETFE film is detached from the LSCIPV, the *η*_edge_ faintly changes from 69.8% to 69.4%. Furthermore, the internal photon efficiency (*η*_int_) is calculated to be 18.1% for both the LSCIPV with and without an ETFE film (see details in Note S8, Supporting Information).^[^ ^47^ ^]^ In fact, we find that parts of radiative photons, which would otherwise escape from the TBRb/OSTE hybrids, can be effectively confined within the ETFE film and redirected toward the edge of the LSCIPV (Figure 3e,f and Note S9, Supporting information). Thus, the presence of the ETFE film reduces the reflection while not corrupting the edge output of the LSCIPV.

To evaluate the photostability of the LSCIPV, extended durability tests were conducted under 10 W UV light exposure (see details in Note S10, Supporting Information), with a temperature range of 22–27 °C and a humidity of 52 ± 5%. As shown in Figure 4a, the results indicate no discernible degradation in the PCE and PL intensity, even after 800 h of UV irradiation. This is further supported by the stable PL of TBRb shown in Figure 4b, which indicates negligible PL change after long‐term UV light exposure. Interestingly, after 800 h of UV light irradiation, the LSCIPV exhibits an increase in the *EQE*_LSC_ band at approximately 330 nm (Figure 4c). However, the calculated $J_{\text{sc}}^{\text{Int}}$ from *EQE*_LSC_(*λ*) remains largely unchanged due to the low UV photon flux in the solar spectrum contributing to the output conversion of the LSCIPV. To verify this fact, a comparison of the optical properties of the LSCIPV before and after the durability tests was conducted (Figure 4d). Transmittance (*T*(*λ*)) spectra below 450 nm are notably affected by prolonged UV irradiation, while *R*(*λ*) spectra exhibit negligible changes. The effect of UV irradiation on the variation of the T(*λ*) spectra below 450 nm becomes negligible after 600 h of exposure (see details in Figure S9, Supporting Information). The characteristic absorption bands of TBRb in the *A*(*λ*) spectra at 490 and 525 nm remained unaltered, indicating that the optical properties of TBRb are not significantly affected by long‐term UV irradiation and relatively high humidity. The stability of TBRb's optical is attributed to the oxygen‐scavenging and moisture‐blocking properties of the OSTE polymers (see details in Figure S10, Supporting Information). Figure 4e compares the *T*(*λ*) spectra of OSTE polymers and LSCIPV before and after the same durability tests. Similar variations in the *T*(*λ*) spectra below 450 nm are observed for both of them. Based on these analyses, it can be deduced that the variations in *EQE*_LSC_, *T*(*λ*) spectra, and *A*(*λ*) spectra are primarily attributed to the changes in the optical properties OSTE polymers.

**Figure 5:**
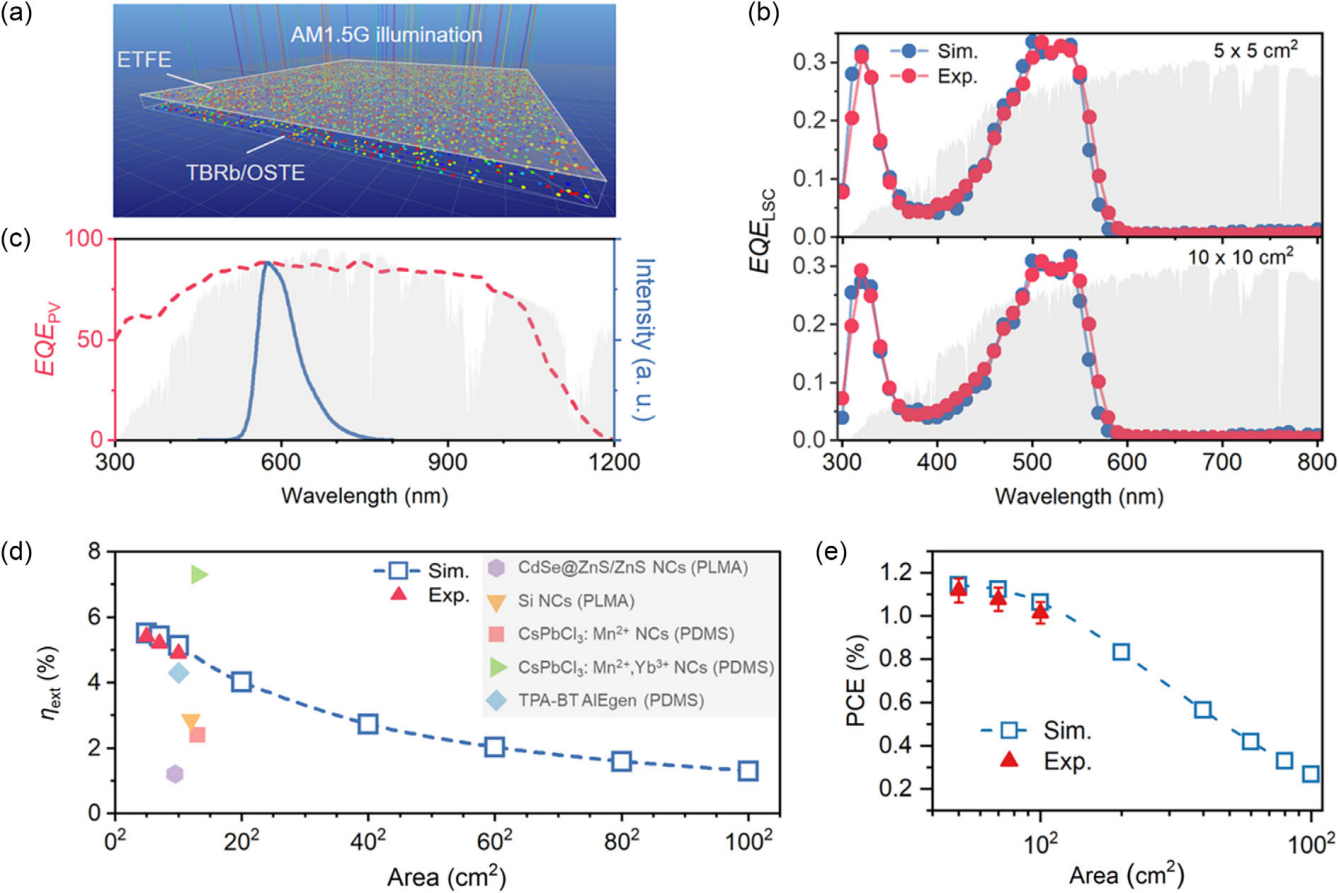
a) Schematic representation of visual Monte Carlo ray‐tracing simulations for *EQE*_LSC_ estimation. b) Simulated and experimentally measured *EQE*_LSC_ spectra of the LSCIPV with different device areas. The AM 1.5 G photon flux spectrum is shown by gray shading. c) The external quantum efficiency (*EQE*_PV_) spectrum of the Si solar cell, the PL spectrum of the LSCIPV, and the AM 1.5 G photon flux spectrum (shaded gray). d) Simulated (blue squares) and experimentally measured (red triangles) *η*_ext_ of the LSCIPV as a function of device areas. Several previous efficiency records of flexible LSCs are plotted for comparison. e) Simulated (blue squares) and experimentally measured (red triangles) PCE of the LSCIPV as a function of device areas.

A Monte Carlo ray‐tracing simulation model with conditions comparable to the practical application scenarios is built to estimate the performances of large LSCIPV potentially achievable, as shown in **Figure** **5**a.^[^ ^48^ ^]^ The model was designed to mimic real‐world scenarios, with vertically incident light illuminating the entire top surface of the LSCIPV model. The AM 1.5 G photon flux solar spectrum was set as the incident light source. The detailed process of the Monte Carlo ray‐tracing simulation is described in Note S13, Supporting Information. Figure 5b shows both the simulated and the experimentally measured *EQE*_LSC_ spectra for the LSCIPV with the area of 5 × 5 and 10 × 10 cm^2^, respectively. The simulated *EQE*_LSC_(*λ*) profile coincides well with the direct experimental measured ones, indicating the feasibility of our simulation model for estimating the performances of large LSCIPV. The *EQE*_LSC_(*λ*) profile is used to calculate the external photon efficiency (*η*_ext_(*λ*)) using the following equation:^[^ ^44^ ^]^

(2) $$EQE_{\text{LSC}}\left(\lambda \right)=\eta_{\text{ext}}\left(\lambda \right)\cdot \frac{\int EQE_{\text{PV}}\left(\lambda^{\prime }\right)\cdot PL\left(\lambda^{\prime }\right)d\lambda^{\prime }}{\int PL\left(\lambda^{\prime }\right)d\lambda^{\prime }}$$

where the integral term represents the *EQE* of the edge‐mounted PV cell over the emission wavelengths of the luminophore (Figure 5c), and *PL*(*λ*′) is the luminophore photoluminescence spectrum in waveguide matrix as a function of wavelength. The overall *η*_ext_ can then be calculated as:

(3) $$\eta_{\text{ext}}=\frac{\int \mathrm{AM}1.5G\left(\lambda \right)\cdot \eta_{\text{ext}}\left(\lambda \right)d\lambda }{\int \mathrm{AM}1.5G\left(\lambda \right)d\lambda }$$

where AM 1.5 G(*λ*) is the AM 1.5 G photon flux solar spectrum ranging from 300 to 1200 nm. As shown in Figure 5d, the measured *η*_ext_ is calculated to be 5.4%, 5.2%, and 4.9% for a device with area of 5 × 5, 7 × 7, and 10 × 10 cm^2^, respectively. These calculated simulated *η*_ext_ values align well with the calculated measured ones. Furthermore, the simulated results show a relatively high *η*_ext_ of 1.3% for a large device area of 1 m^2^. In comparison to flexible LSCs based on NCs or aggregation‐induced‐emission luminogens (AIEgens),^[^ ^11^, ^12^, ^13^, ^49^ ^]^ *η*_ext_ of our flexible LSCIPV falls within the upper‐middle‐range among these previously reported flexible LSCs (see details in Table S3, Supporting Information).

## 3 Conclusion

In conclusion, TBRb/OSTE hybrids are developed for LSC applications, where OSTE polymers act as oxygen scavengers to enhance the photostability of TBRb, increase the Stokes shift in TBRb by altering the dipole moment, and improve the *Φ*_PL_ of TBRb by impeding nonradiative intramolecular motions. Moreover, we propose a concept of LSCIPV by preparing and coupling LSC with the silicon solar cell directly, and encapsulating both components as a single unit. Building upon the excellent luminescence properties of TBRb/OSTE hybrids, the flexible LSCIPV achieves a PCE of 1.04% through an AR effect and possesses a hydrophobic surface for self‐cleaning purposes. Furthermore, the LSCIPV exhibits long‐term photostability. Monte Carlo ray‐tracing simulations indicate that an *η*_ext_ of 1.3% can be attained for 1 m^2^ device. This work not only develops TBRb as promising luminophores for flexible LSCs, offering flexible power generation with high photovoltaic performance, and long‐term photostability, but also introduces the concept of LSCIPV, simplifying the preparation process for deploying LSCs as power‐generating sources.

## 4 Experimental Section

4.1

4.1.1

##### Chemicals and Materials

Chemical reagents tetra(t‐butyl)rubrene and N, N‐dimethylformamide were purchased from Ossila. The pentaerythritol tetrakis(3‐mercaptopropionate) (thiol) (>90%), triallyl triazine trione (allyl) (98%), and photo‐initiator (Irgacure‐184) (98%) were purchased from Aladdin.

*Synthesis of TBRb/OSTE hybrids:* A highly concentrated solution was prepared by dissolving 0.01 g of TBRb in 2   mL of toluene and 0.5 mL of N, N‐dimethylformamide. This TBRb solution was then added dropwise to 55 mL of the precursor, which consisted of a mixture of thiol monomers, allyl monomers, and Irgacure‐184 at a mass ratio of 1:1:0.01, under vigorous stirring. After stirring vigorously for 15 min, the mixture was placed in a vacuum chamber at −0.1 MPa for 0.5 h to remove any bubbles. Following the vacuum treatment process, the mixture was transferred into a quartz bottle. The quartz bottle was then irradiated with 6 W UV light for 10 min under vacuum.

##### Preparation of LSCIPVs

A laser‐cut silicon solar cell (10 × 0.5 cm^2^, see Note S15, Supporting Information) was affixed to a TPT backsheet using hot melt EVA under pressure at 80 °C for 20 min. An ETFE film was placed on the inner bottom surface of the glass mold (internal dimensions of 10 × 10.2 × 0.5 cm^3^). The cell was then placed upright on the film, close to the inner surface of the mold. A highly concentrated solution was prepared by dissolving 0.01 g of TBRb in 2  mL of toluene and 0.5 mL of N, N‐dimethylformamide. This TBRb solution was then added dropwise to 55mL of the precursor, which consisted of a mixture of thiol monomers, allyl monomers, and Irgacure‐184 at a mass ratio of 1:1:0.01, under vigorous stirring. After stirring vigorously for 15 min, the mixture was placed in a vacuum chamber at −0.1 MPa for 0.5 h to remove any bubbles. Following the vacuum treatment process, the mixture was transferred into the glass mold. The entire preparation platform was irradiated by 6 W UV light for 10 min under vacuum. Finally, the glass mold was detached, and the LSCIPV was obtained.

##### Optical Characterizations

The PL spectra and time‐resolved PL decay curves were measured in a fluorescence spectrophotometer (Edinburgh Instrument Ltd., FLS1000) equipped with a xenon lamp (450 W) and a pulse 460 nm laser as the excitation sources. Temperature‐dependent PL decay curves were recorded on the FLS1000 spectrophotometer using a pulse 460 nm laser as an excitation source and the samples were mounted on a thermal stage (FTIR600, Linkam Scientific Instruments). The *Φ*_PL_ was measured using the integrated sphere internally installed in the FLS1000 instrument. The *η*_edge_ of LSCIPVs was measured using an integrating sphere fiber coupled with the FLS1000 instrument. *T* and *R* spectra were measured using a double‐beam Shimadzu UV‐3600 spectrophotometer with its integrating sphere. Optical microscopy photographs were performed using the metallographic microscope (Soptop CX40M, Shunyu). Digital photographs were captured by a camera phone (Galaxy S21 Ultra 5 G, Samsung).

##### Photovoltaic Characterization

The four edges of the LSCIPV were covered with black tapes to block the light and internal reflection of light. A matte black background was placed on the back of the LSCIPV to eliminate illumination from the environment or reflection. The *J*–*V* characteristic was measured using PVIV‐412 V test station (Newport, USA) under simulated AM 1.5 G solar illumination (see Note S16, Supporting Information). For *EQE*_LSC_ measurements, a light blocker with an opening was closely placed in front of the LSCIPV to block any direct illumination from the environment. The incident beam travels through the opening of the light blocker and then irradiates to the surface of the LSCIPV. The measured *EQE*_LSC_ at each distance (*d*) was corrected by multiplying the geometric factor *g = π/tan* ^−1^ *(L*/2* d)*, which accounts for the different angle subtended by the edge‐mounted PV at various excitation distance (*d*), where L is the LSCIPV length.

##### Monte Carlo Ray‐Tracing Simulation

Theoretical analysis of the *EQE*_LSC_ of the LSCIPV was performed using a Monte Carlo ray‐tracing method based upon Python software, in which propagation of a photon within the LSCIPV was modeled as the propagation of a geometrical ray subject to refraction/reflection at the interfaces according to Fresnel laws. The stochastic nature of the simulations is reflected in the fact that the ray is not split upon reaching an interface but is instead transmitted or reflected, with probabilities proportional to the respective energy fluxes given by the Fresnel laws (see Note S13, Supporting Information).

## Conflict of Interest

The authors declare no conflict of interest.

## Supporting information

Supplementary Material

## Data Availability

The data that support the findings of this study are available from the corresponding author upon reasonable request.
