# Supplementary material for: Engineering Highly Emissive Tetra(t‐Butyl)rubrene/off‐Stoichiometry Thiol‐Ene Hybrids Toward Flexible Luminescent Solar Concentrator‐Integrated Photovoltaics with Excellent Stability
Source: Small Sci. 2024 Apr 5;4(12):2400121. doi: 10.1002/smsc.202400121 (PMC11935255; doi:10.1002/smsc.202400121)
Supplement: Supplementary file 1 — Supplementary Material [file SMSC-4-2400121-s001.pdf]

Engineering highly emissive tetra(t-butyl)rubrene/off-stoichiometry thiol-ene hybrids towards flexible luminescent solar concentrator integrated photovoltaics with excellent stability

Yi Zhang, Zida Zheng, Zhixing Gan, Rui Huang,* and Xiaowei Zhang**

Dr. Y. Zhang, Dr. Z Zheng, Prof. X. Zhang

Department of Electrical Engineering and Computer Science

Ningbo University

Ningbo, Zhejiang 315211, China.

E-mail: zhangxiaowei@nbu.edu.cn

Prof. Z. Gan

Center for Future Optoelectronic Functional Materials

Nanjing Normal University

Nanjing, Jiangsu 210093, China

E-mail: zxgan@njnu.edu.cn

Prof. Z. Gan, Prof. X. Zhang

National Laboratory of Solid State Microstructures

Nanjing University

Nanjing, Jiangsu 210093, China

Dr. Y. Zhang, Prof. R. Huang,

School of Materials Science and Engineering

Hanshan Normal University

Chaozhou, Guangdong 521041, China

E-mail: rhuang@hstc.edu.cn

Note S1. The thiol-ene click reaction on OSTE/TBRb hybirds

A typical thiol-ene polymer is composed of two types of monomers (Figure S1a): one with thiol functional groups, represented as $xR_1\text{–}(SH)_4$, and the other with allyl functional groups, represented as $yR_2\text{–}(CH_2\text{–}CH{=}CH_2)_3$. In these representations, “x” and “y” denote the number of monomers of each type. In off-stoichiometry thiol-enes (OSTE) polymers, off-stoichiometric formulations of thiol-enes with an excess of allyl functional groups (where $4x < 3y$) result in a polymer with residual unreacted allyl anchor groups (Figure S1b).

Figure S2 shows the FTIR spectra of the TBRb and OSTE mixtures (before UV curving) and the TBRb/OSTE hybrids (after UV curving). The band at 3490 cm^{-1} is attributed to the -OH stretching vibration absorption of Irgacure 184.[1] After UV curing, the disappearance of the band at 3490 cm^{-1} indicates that Irgacure 184 was fully consumed in the reaction. A new band emerges at 663 cm^{-1} after UV curing, corresponding to the C-S-C symmetric stretch of the thiol-ene crosslink.[2] No other change is observed. Thus, we infer that no radicals were generated. This conclusion is further corroborated by the negligible change in the absorption profile of the TBRb before and after UV curing, as shown in Figure 1.

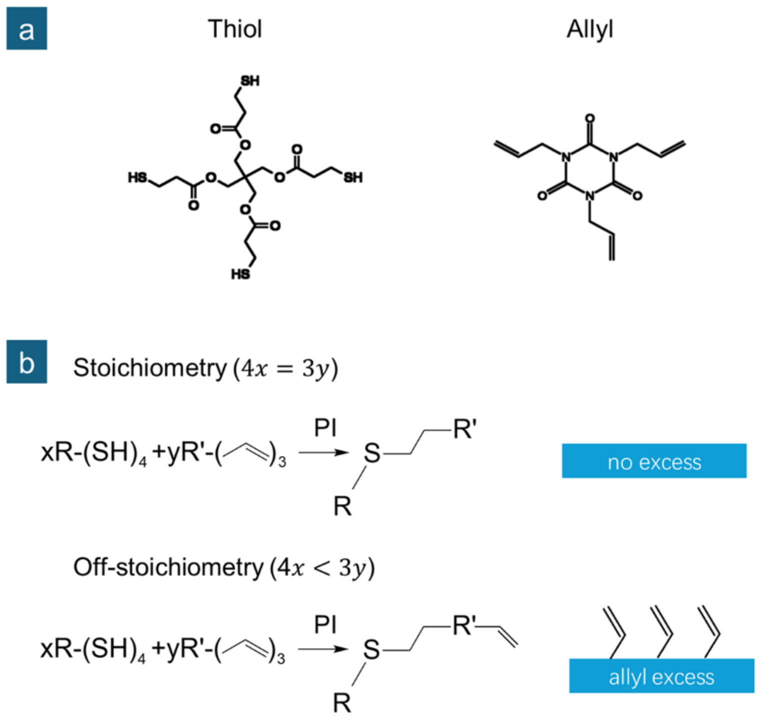

Figure S1 a) The molecular structure of thiol and allyl monomers. b) Standard stoichiometric and Off-stoichiometry formulations of thiol–ene systems.

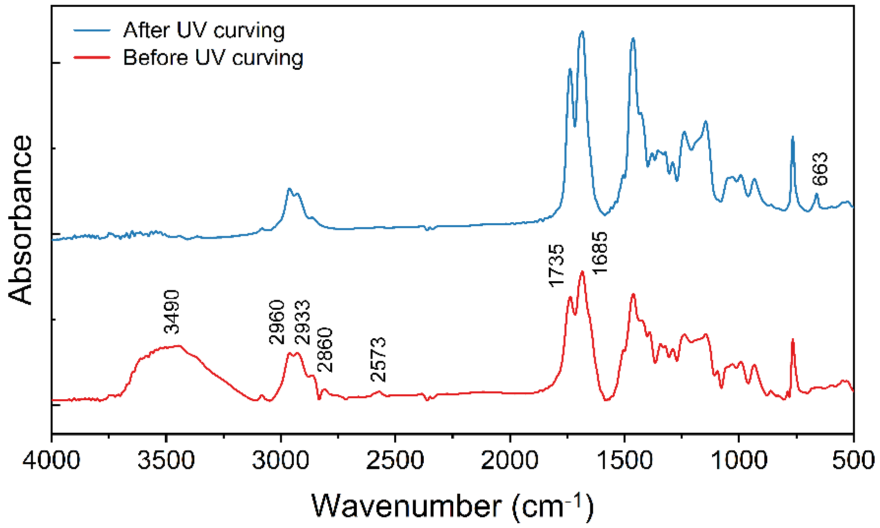

Figure S2 The FTIR spectra of the TBRb and OSTE mixtures (before UV curving) and the TBRb/OSTE hybrids (after UV curving).

Note S2. Calculation and comparison of modified overlap integrals

We adopt the overlap integral (OI*) method, as proposed by Lunt and co-workers, for comparison with other luminophores. OI* is defined as:

$$\mathrm{OI}^* = \frac{\int_0^\infty A(\lambda)\cdot PL^*(\lambda)d\lambda}{\int_0^\infty PL^*(\lambda)d\lambda}$$

where $A(\lambda)$ and $PL^*(\lambda)$ represent the absolute absorption and normalized PL spectra of the luminescent material, respectively.

Table S1 OI* for the LSCs based on different types of luminophores.

Luminophore	OI*	Ref.
TBRb/OSTE	0.028	This work
$Cs_2Mo_6I_8(CF_3CF_2COO)_6$	0.015	Adv. Energy Mater. 2021, 11, 2003581.[3]
COi8DIFC	0.40	
BODIPY	0.56	
Cy7-NHS	0.47	Adv. Optical Mater. 2020, 1901536.[4]
AIEgen TPP1	0.055	Dyes and Pigments 202 (2022) 110221.[5]
$NBDNH_2$	0.123	Dyes and Pigments 222 (2024) 111869.[[6]

As reported by Richards et al., an overlap integral (OI*) value of 0.028 in their designed luminophore will indeed limit a PCE of only ~1% at the scale of 100,000 cm².[7] However, it's important to note that the efficiency of an LSC depends on but not limited to OI*. Other factors are also very crucial, including the match degree between the PL of the luminophore and the quantum efficiency of the PV cell, the absorbance and absorption range of the luminophore, the specific overlap profiles between the absorption and PL spectra of the luminophore, and so on. The OI* is a metric used for evaluating self-absorption losses in devices. In a recent work, CIS/ZnS QDs with OI* > 0.05 were used to construct the LSCs with the highest device efficiency ever recorded.[8]

Note S3. Luminophores for LSCs.

Table S2 Photoluminescence quantum yield and absorption cross-section of typical luminophores.

**Luminophores**	**Φ_{PL} (%)**	**Absorption cross-section at 495 nm (cm^2)**	**Ref.**
$CuInS_2/ZnS$	72	2.4×10^{-15}	*Nat Photon.,* 2018, 12,105–110.[9]
C NCs	65	1.7×10^{-17}	*Energy Environ. Sci.*, 2021, 14, 396-406.[10]
Si NCs	50	1.5×10^{-16}	*Nat Photon.,* 2017, 11, 177-185.[11]
PbS/CdS	40-50	9.6×10^{-16}	*Adv. Energy Mat.,* 2016, 6, 1501913.[12]
CdSe/CdS	45	2.8×10^{-15}	*Nat. Photon,* 2014, 8, 392-399.[13]
**TBRb**	**89**	**3.1×10^{-14}**	**This work**

Note S4. Temperature-dependent time resolved photoluminescence

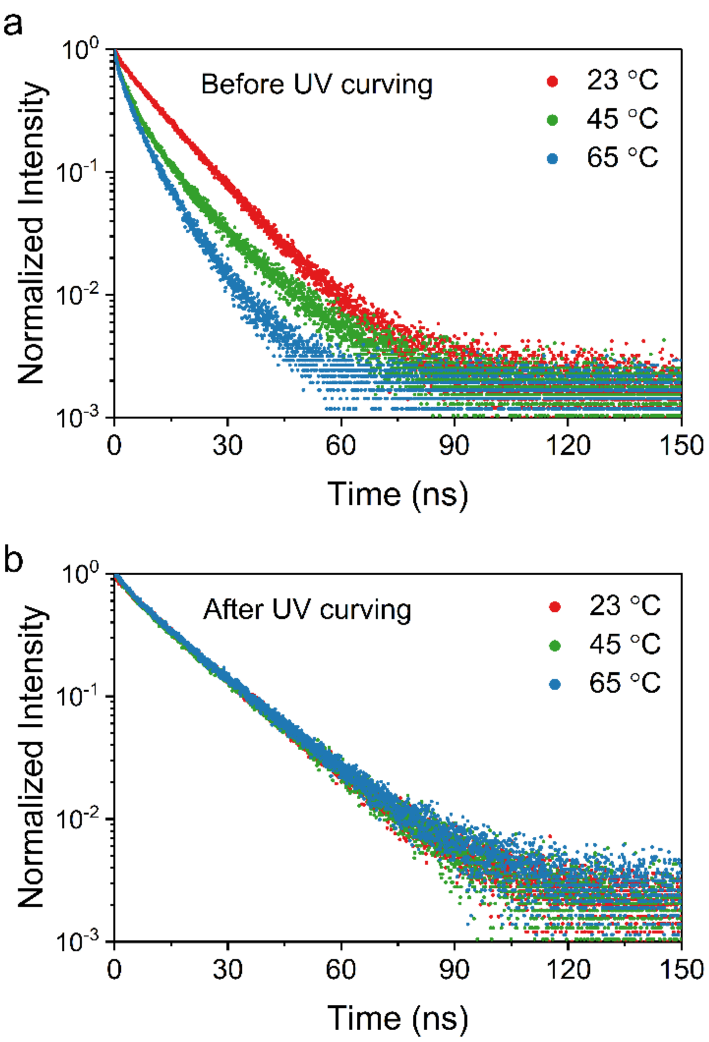

Figure S3. Temperature-dependent PL decay curves of the a) TBRb and OSTE mixtures and b) the TBRb/OSTE hybrids obtained at an excitation wavelength of 460 nm monitoring at 540 nm.

Note S5. Flexural characteristics of LSCIPVs

The LSCIPV can be easily bent from flat ($\theta = 0°$) to semi-cylindrical ($\theta = 45°$) configurations, as shown in Figure S4a-b. Figure S4c demonstrates that after 300 semi-cylindrical bends, the PCE of the LSCIPV marginally decreased from 1.04 ± 0.05% to 0.97 ± 0.05%. No looseness was detected in the coupling of the solar cell to the LSC (Figure S4d-e).

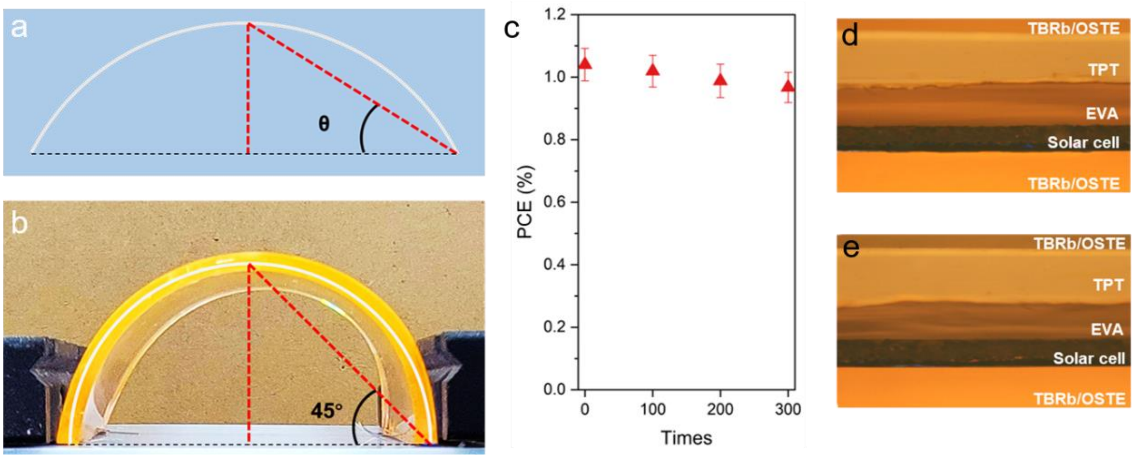

Figure S4. a) Schematic representation of an LSCIPV with θ. b) Photograph of an LSCIPV bent to a semi-cylindrical configuration. c) PCE of the LSCIPV plotted as a function of bending times. A confocal microscope image of the internal composition of the LSCIPV d) before bending and e) after 300 bending cycles.

Note S6. Average visible transmittance

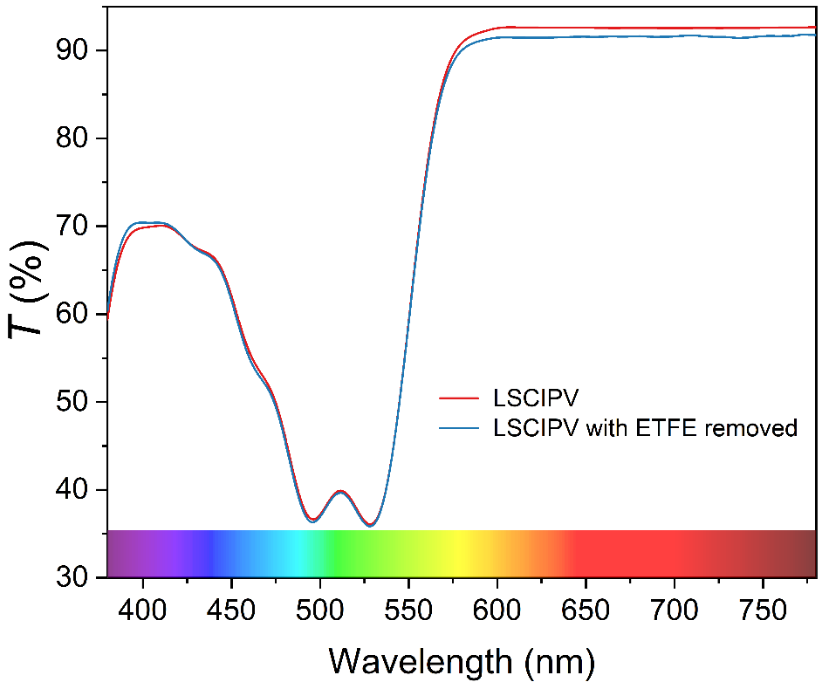

Figure S5. Transmittance spectra for the LSCIPV and the same one without the ETFE film.

Note S7. Analytic model of the anti-reflection effect

We consider the incident light with a wavelength of 600 nm, which is minimally absorbed by the ETFE film, TBRb molecules, and OSTE polymers. Consequently, absorption events within the media can be disregarded. When the incident lights irradiate on the device, the light beam experiences multiple reflection and transmission events at the interface between two media with different refractive indexes. The reflectance at the interface is calculated as:

$$R_{0-1} = \frac{(n_1 - n_0)^2}{(n_1 + n_0)^2}$$

where n_0 is the refractive index of the medium from which the light originates, n_1 is the refractive index of the medium to which the light is directed. The n is 1 for air (n_A), 1.4 for the ETFE film (n_E) and 1.53 for OSTE polymers (n_O).

As shown in Figure S1a, for the LSCIPV without the ETFE film, the reflectance event ① occurring at the air-OSTE interface is calculated:

$$R_1 = R_{A-O}$$

The reflectance event ② occurring at the OSTE-air interface is calculated:

$$R_2 = (1 - R_{A-O}) \times R_{A-O} \times (1 - R_{A-O})$$

The total reflectance (R) for the LSCIPV without the ETFE film is calculated:

$$R = R_{A-O} + (1 - R_{A-O}) \times R_{A-O} \times (1 - R_{A-O}) = 8.4\%$$

As for the LSCIPV shown in Figure S1b, the reflectance event ① occurring at the air-ETFE interface is calculated:

$$R_1 = R_{A-E}$$

The reflectance event ② occurring at the ETFE-OSTE interface is calculated:

$$R_2 = (1 - R_{A-E}) \times R_{E-O} \times (1 - R_{A-E})$$

The reflectance event ③ occurring at the OSTE-air interface is calculated:

$$R_3 = (1 - R_{A-E}) \times \left(1 - R_{E-O}\right) \times R_{A-O} \times (1 - R_{E-O}) \times (1 - R_{A-E})$$

The total reflectance (R) for the LSCIPV is calculated:

$$R = R_{A-E} + (1 - R_{A-E}) \times R_{E-O} \times (1 - R_{A-E}) + (1 - R_{A-E}) \times \left(1 - R_{E-O}\right) \times R_{A-O} \times (1 - R_{E-O}) \times (1 - R_{A-E}) = 7.1\%$$

The calculated R values coincide with the experimentally measured results in Figure 4c.

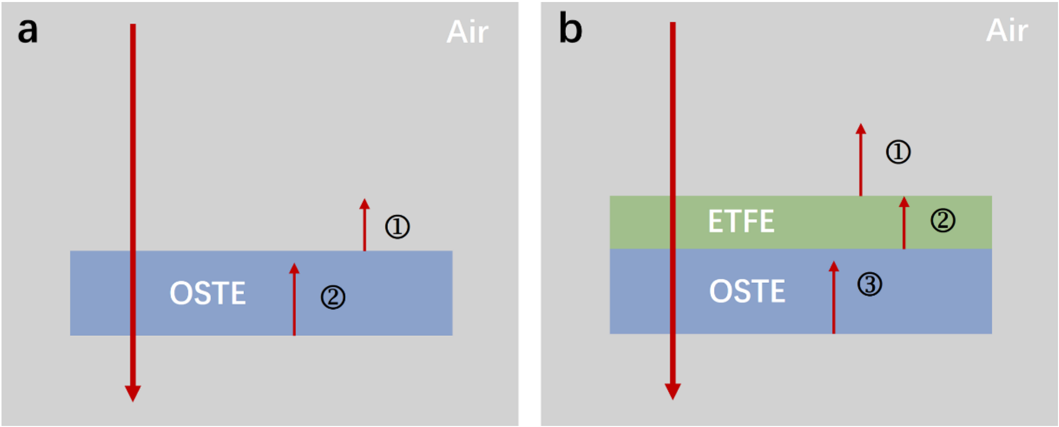

Figure S6. Diagram of the incident light beam experiences reflection events for a) the LSCIPV without the ETFE film and b) the LSCIPV.

Note S8. The calculation of internal photon efficiency

The internal photon efficiency (η_{int}), as defined by Debije et al., is given by:[14]

$$\eta_{int} = \frac{\text{no. of edge} - \text{emitted photons}}{\text{no. of total absorbed photons}}$$

$$= \frac{\text{no. of edge} - \text{emitted photons}}{\text{no. of total incident photons}} \times \frac{\text{no. of total incident photons}}{\text{no. of total absorbed photons}}$$

$$= \eta_{ext} \times \frac{1}{\eta_{abs}}$$

According to the calculated η_{ext} presented in the manuscript, η_{ext} is 4.9% for the LSCIPV and 4.6% for the LSCIPV without an ETFE film. η_{abs} can be calculated from the quasi-absorption spectra in Figure 3c. η_{abs} is 27% for the LSCIPV and 26% for the LSCIPV without an ETFE film. Consequently, η_{int} is calculated to be 18.1% for both the LSCIPV and the LSCIPV without an ETFE film.

Note S9. Analyses of light trapping

To find the fraction of luminescent photons trapped by total internal reflection at the front surface of the device, we solve Snell's law as in Figure S2. Consider a photon radiated at the critical angle (θ_c), the angle of incidence greater than for which the photon is internally reflected at the interface. Following Snell's law, θ_c is as follows:

$$\theta_c = arcsin\, {}^{n_0}/_{n_1}$$

#(9) n_1 is the refractive index of the medium where radiated photons in, n_0 is the refractive index of the other medium. As shown in Figure S2a, the θ_c at the air/OSTE interface is calculated:

$$\theta_c = arcsin({}^{n_{air}}/_{n_{OSTE}}) = 40\,°8'$$

where n_{air} is the refractive index of air, n_{OSTE} is the refractive index of OSTE polymers. Luminescent photons with an angle of incidence greater than 40°48' are trapped in the LSCIPV with the ETFE film removed.

In the LSCIPV (see Figure S2b), the θ_c at the ETFE/OSTE interface is calculated:

$$\theta_c = arcsin({}^{n_{ETFE}}/_{n_{OSTE}}) = 66°12'$$

where n_{ETFE} is the refractive index of the ETFE film. This implies that a higher number of luminescent photons can escape from the ETFE/OSTE interface compared to the air/OSTE interface. Specifically, the additional photons that escaped have an angle of incidence ranging from greater than 40°48' to less than 66°12'. Following Snell's law, luminescent photons escaped from OSTE polymers to the ETFE film with the angle of refraction (θ_2) is:

$$\theta_2 = \frac{sin\theta_1 \cdot n_O}{n_E}$$

where θ_1 is the angle of incidence, n_O is the refractive index of the OSTE polymers, and n_E is the refractive index of the ETFE film (Figure S2b). The luminescent photons that escape with θ_1 of 40°48′ corresponded to θ_2 of 45°39′, and the θ_c at the air/ETFE interface is calculated:

$$\theta_c = arcsin({}^{n_{air}}/_{n_{ETFE}}) = 45°35'$$

The lowest θ_2 of 45°39′ is greater than the θ_c of 45°35′ at the air/ETFE interface. This suggests that the additional luminescent photons that escaped are trapped within the ETFE film. Consequently, the edge emission efficiency (η_{edge}) of the LSCIPV remains unchanged when compared to the LSCIPV with the ETFE film removed. These findings align with the observations shown in Figure 4e, f.

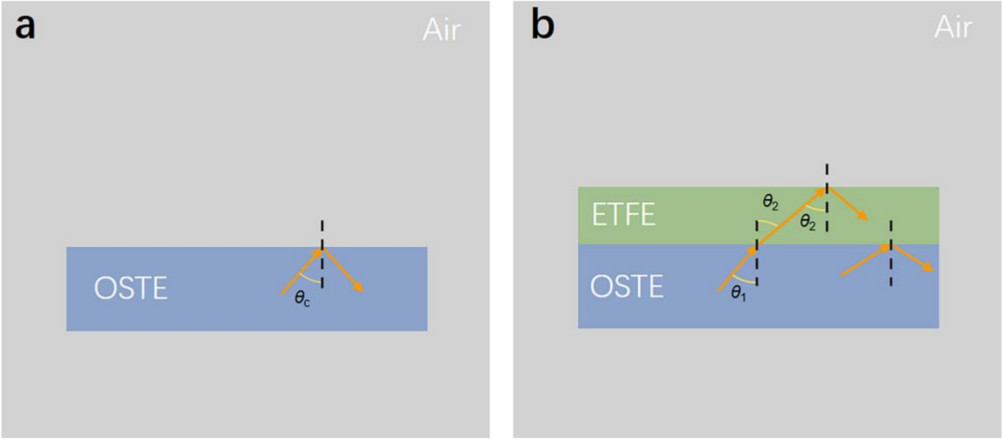

Figure S7. Diagram of the refraction and total internal reflection of luminescent photons for a) the LSCIPV without the ETFE film and b) the LSCIPV.

Note S10. Experimental conditions for UV photostability test.

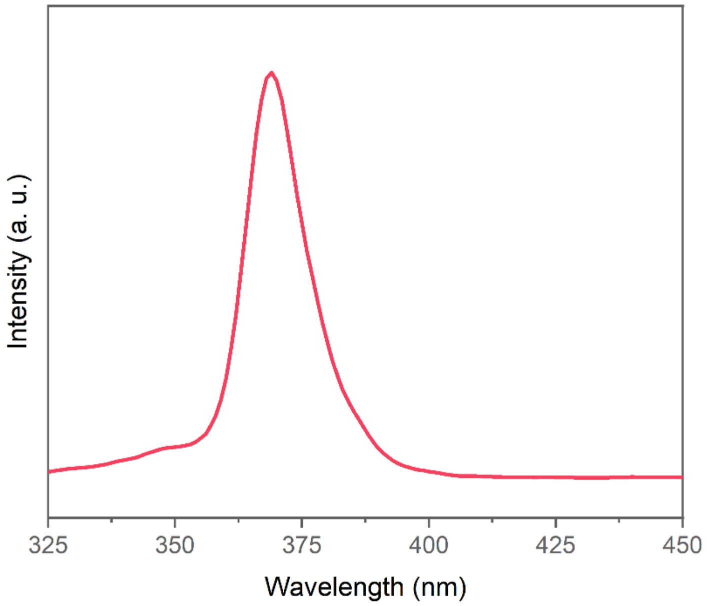

Figure S8. Emission spectrum of the UV lamp. The radiance of UV illumination on the surface of the LSCIPV is 2.8 W m^{-2}.

Note S11. Stability of transmittance spectra for LSCIPV

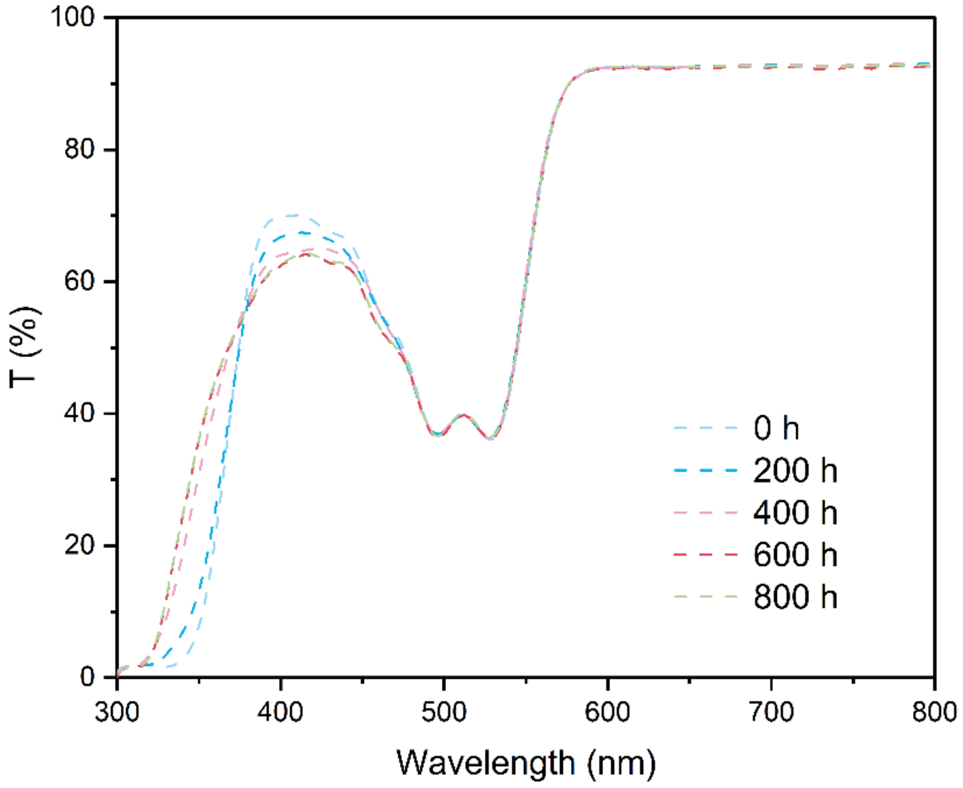

Figure S9. T(λ) spectra for the LSCIPV as a function of UV light exposure time.

Note S12. Gas permeability tests for TBRb/OSTE hybrids

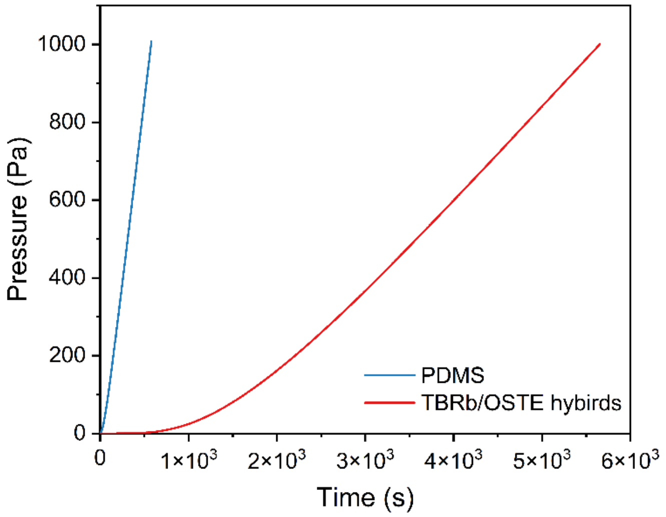

Figure S10 Pressure plotting to time from O_2 gas permeability measurements. The gas permeability coefficients of TBRb/OSTE hybrids and the PDMS film are deduced to be 4.08×10^{-10} and 3.44×10^{-9} $cm^3\cdot cm\cdot cm^{-2}\cdot s^{-1}\cdot cmHg^{-1}$, respectively.

Note S13 Monte Carlo ray tracing simulations

Monte Carlo ray-tracing simulation is an approach of applying repeated random sampling to determine the fate of many simulated rays within an LSCIPV. The outcomes of many events encountered by rays within the LSCIPV are determined using random number generation weighted by calculated probabilities based on the physical properties of the system. Probabilities are assigned to light absorption, emission, non-radiative recombination, scattering, reflection and transmission from TBRb, OSTE polymers and the ETFE film (Figure S11). Simulation input parameters include the number of incident photons (400,000), wavelengths of incident light, the position and direction of the incident light, dimensions of the LSC, refractive indexes of OSTE polymers, the ETFE film and air, the photoluminescence quantum yield of TBRb, the PL spectrum of TBRb, the absorption coefficient of the ETFE film, OSTE polymers and TBRb, scattering coefficient, and anisotropy factor.

In this simulated model, LSCIPV components have been broken up into several general categories. The LSCIPV geometry consists of the following classes of objects (Figure S12):

Boundary objects: There are three types of boundaries containing outer boundaries, inner boundaries and collected boundaries. The boundary is used to determine the behavior of the angle between the propagation direction of the photon and the boundary constrained by Snell's Law.

Particle objects: A particle object is assigned to a particle volume to handle light interaction with TBRb molecules. Light interactions contain absorption, emission and scattering events, and the characteristics of these events are traced.

Interior objects: A interior object is used to determine the fate of scattering and absorption elements of light without encountering the particle object in the OSTE polymers and the ETFE film. Light propagation in the OSTE polymers or the ETFE film will inherit some characteristics of light before these events. For example, the reflected photon will inherit the random free path of the photon before reflection, but it changes the propagation direction of the photon.

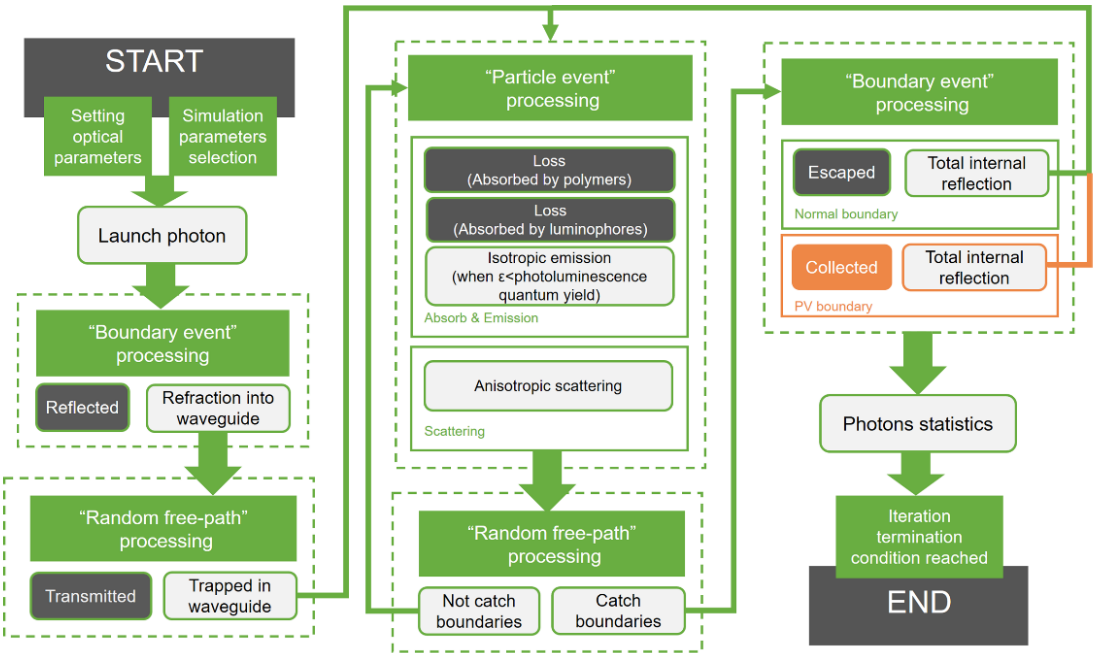

Figure S11. Illustration of Monte Carlo ray-tracing simulated path in the LSCIPV. Logic flow chart of the Monte Carlo ray-tracing simulation for LSCIPVs.

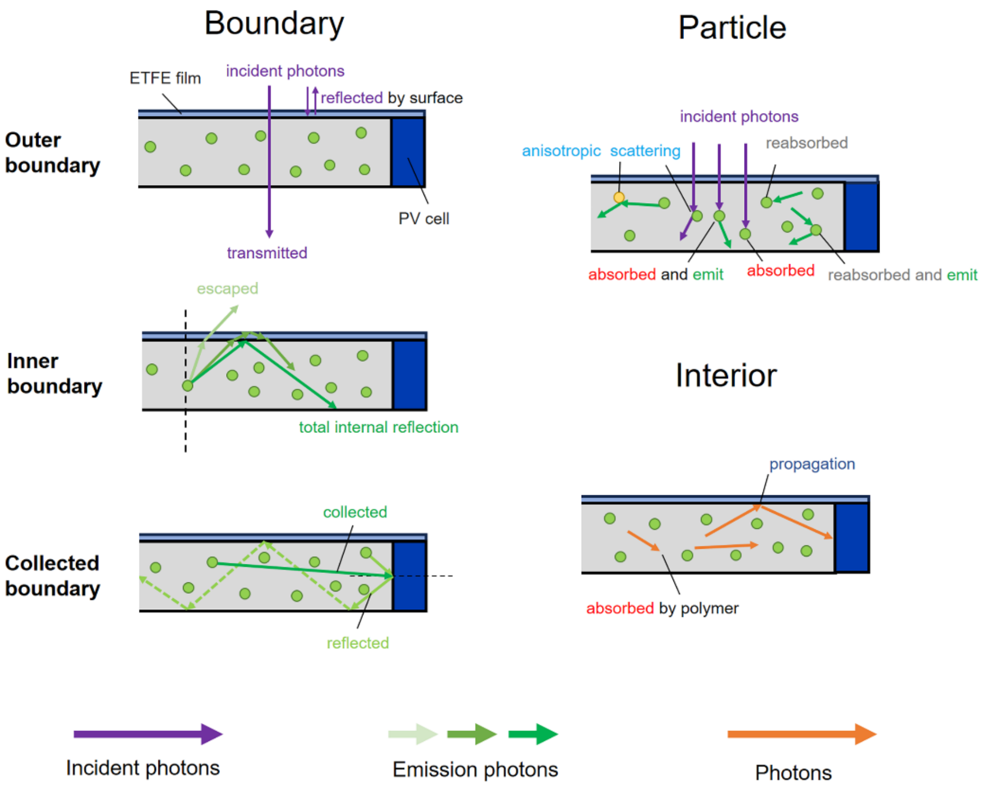

Figure S12. Illustration of Monte Carlo ray-tracing simulated model in the LSCIPV. A schematic of three classes of objects (boundary, particle and interior in LSCIPVs) and light interactions with various objects.

Note S14. Summary of literature reports

Table S3. An overview of literature reports for flexible LSCs.

Luminophore(s)	Φ_{PL} (%)	Area (cm^2)	AVT (%)	PCE (%)	G[a]	References
Si NCs	46±5	144	73	2.85	11.5	[11]
CdSe@ZnS/ZnS NCs	79-83	90	84.4	0.34	11.8	[15]
Mn^{2+}-doped $CsPbCl_3$ NCs	48.4	169	-	0.29[b]	6.3	[16]
Mn^{2+}/Yb^{3+} codoped $CsPbCl_3$ NCs	125.3	169	-	1.69[b]	6.3	
TPA-BT AIEgen	~100	100	87	1.4[c]	12.5	[17]
This work	89	100	75.3	1.04	5	

[a] *G* is the geometric factor, which is the ratio of the top area (A_{top}) and the edge area (A_{edge}).
[b] The PCE of the solar cell used to coupled LSC device is 8.07%.
[c] The PCE of the solar cell used to coupled LSC device is 18.7%.

The PCE of the LSCIPV system is dependent not only on the optical conversion efficiency of the LSC but also on the PCE of the Si solar cells. To integrate the Si solar cell into the LSCIPV system, we use 10 × 0.5 cm^2 of Si solar cells which are laser cut from a 12.5 × 12.5 cm^2 Si solar cell, instead of using commercial Si solar cells as done in other works.[18–20] These laser-cut solar cells only have a PCE of 14.1 ± 0.6%. We would like to emphasize that the PCE of the laser-cut solar cells is significantly lower than that of current commercial silicon solar cells, which is about 25%. If customized solar cells with PCE of 25% can be produced to fit the LSCIPV system, it is anticipated that the PCE of this LSCIPV system could be significantly improved.

Note S15. Laser-cut silicon solar cells

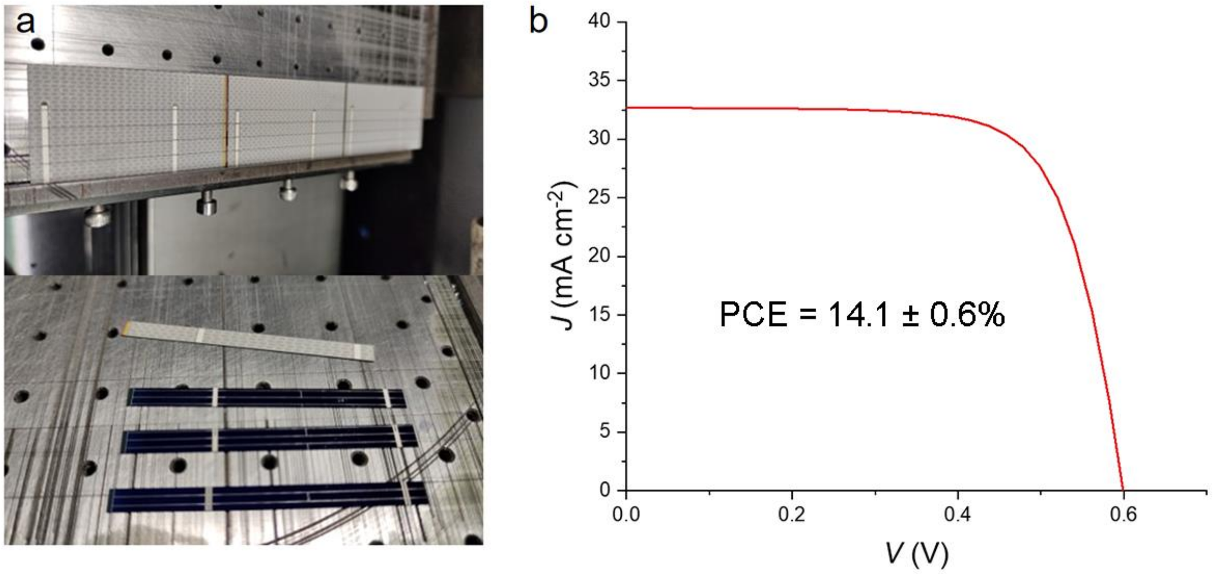

Figure S13 a) Photographs of laser-cut Si solar cells. b) The current density versus voltage (J–V) characteristic of a laser-cut Si solar cell.

Note S16. The solar simulator used in *J–V* measurement.

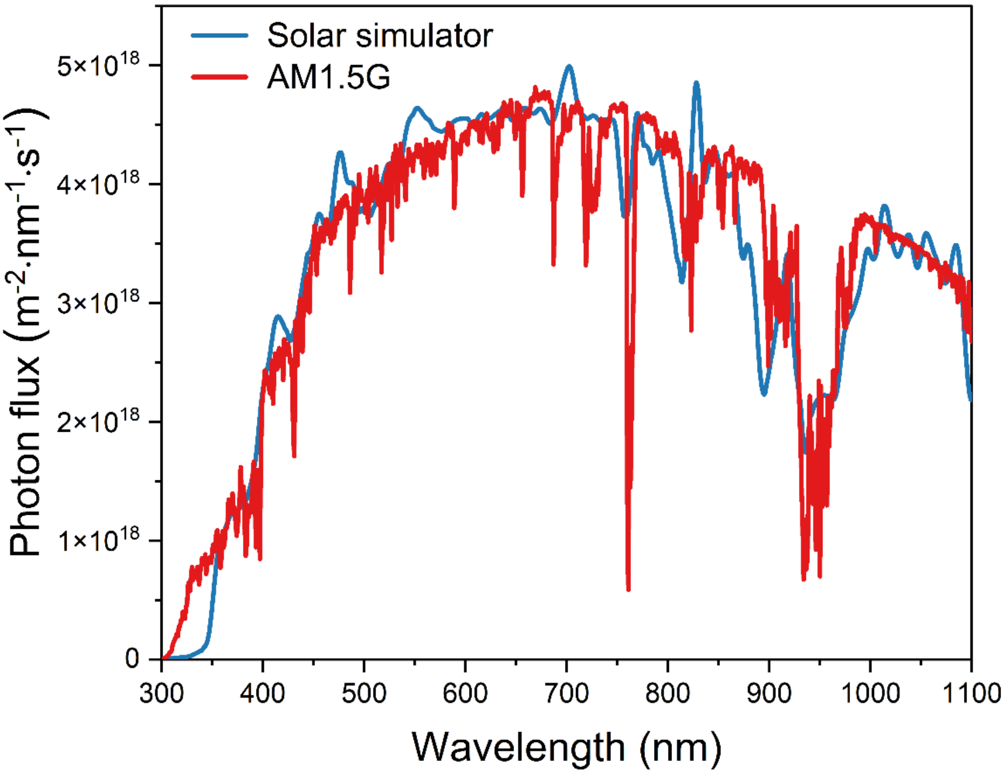

Figure S14 The photon flux spectra of AM 1.5G and the solar simulator.

References

[1] L. Deng, L. Tang, J. Qu, *Prog. Org. Coat.* **2020**, *141*, 105546.
[2] W. R. Fawcett, A. A. Kloss, *Journal of the Chemical Society, Faraday Transactions* **1996**, *92*, 3333.
[3] C. Yang, W. Sheng, M. Moemeni, M. Bates, C. K. Herrera, B. Borhan, R. R. Lunt, *Adv. Energy Mater.* **2021**, *11*, 2003581.
[4] C. Yang, M. Moemeni, M. Bates, W. Sheng, B. Borhan, R. R. Lunt, *Adv. Opt. Mater.* **2020**, *8*, 1901536.
[5] P. Meti, F. Mateen, D. Y. Hwang, Y.-E. Lee, S.-K. Hong, Y.-D. Gong, *Dyes Pigm.* **2022**, *202*, 110221.
[6] A. Kathiravan, F. Mateen, P. Gopinath, D.-Y. Hwang, S.-K. Hong, S. M. H. Qaid, *Dyes Pigm.* **2024**, *222*, 111869.
[7] B. S. Richards, I. A. Howard, *Energy Environ. Sci.* **2023**, *16*, 3214.
[8] K. Park, J. Yi, S.-Y. Yoon, S. M. Park, J. Kim, H.-B. Shin, S. Biswas, G. Y. Yoo, S.-H. Moon, J. Kim, M. S. Oh, A. Wedel, S. Jeong, H. Kim, S. J. Oh, H. K. Kang, H. Yang, C. J. Han, *Nat. Photonics* **2024**, *18*, 177.
[9] K. Wu, H. Li, V. I. Klimov, *Nat. Photonics* **2018**, *12*, 105.
[10] H. Zhao, G. Liu, S. You, F. V. A. Camargo, M. Zavelani-Rossi, X. Wang, C. Sun, B. Liu, Y. Zhang, G. Han, A. Vomiero, X. Gong, *Energy Environ. Sci.* **2021**, *14*, 396.
[11] F. Meinardi, S. Ehrenberg, L. Dhamo, F. Carulli, M. Mauri, F. Bruni, R. Simonutti, U. Kortshagen, S. Brovelli, *Nat. Photonics* **2017**, *11*, 177.
[12] Y. Zhou, D. Benetti, Z. Fan, H. Zhao, D. Ma, A. O. Govorov, A. Vomiero, F. Rosei, *Adv. Energy Mater.* **2016**, *6*, 1501913.
[13] F. Meinardi, A. Colombo, K. A. Velizhanin, R. Simonutti, M. Lorenzon, L. Beverina, R. Viswanatha, V. I. Klimov, S. Brovelli, *Nat. Photonics* **2014**, *8*, 392.
[14] M. G. Debije, R. C. Evans, G. Griffini, *Energy Environ. Sci.* **2021**, *14*, 293.
[15] L. J. Brennan, F. Purcell-Milton, B. McKenna, T. M. Watson, Y. K. Gun'ko, R. C. Evans, *J. Mater. Chem.A* **2018**, *6*, 2671.
[16] T. Cai, J. Wang, W. Li, K. Hills-Kimball, H. Yang, Y. Nagaoka, Y. Yuan, R. Zia, O. Chen, *Adv. Sci.* **2020**, *7*, 2001317.
[17] X. Li, J. Qi, J. Zhu, Y. Jia, Y. Liu, Y. Li, H. Liu, G. Li, K. Wu, *J. Phys. Chem. Lett.* **2022**, *13*, 9177.
[18] H. Zhao, Y. Zhou, D. Benetti, D. Ma, F. Rosei, *Nano Energy* **2017**, *37*, 214.
[19] M. Wei, F. P. G. de Arquer, G. Walters, Z. Yang, L. N. Quan, Y. Kim, R. Sabatini, R. Quintero-Bermudez, L. Gao, J. Z. Fan, F. Fan, A. Gold-Parker, M. F. Toney, E. H. Sargent, *Nat. Energy* **2019**, *4*, 197.
[20] Z. Li, A. Johnston, M. Wei, M. I. Saidaminov, J. Martins de Pina, X. Zheng, J. Liu, Y. Liu, O. M. Bakr, E. H. Sargent, *Joule* **2020**, *4*, 631.
